# Diagnostic and Therapeutic Use of Botox for Breast Reconstruction

**DOI:** 10.26502/acmcr.96550419

**Published:** 2021-10-29

**Authors:** Irene T Ma, Pooja Yesantharao, Halley M Darrach, Jennifer G Seither, Hui He, Dung H Nguyen

**Affiliations:** Division of Plastic Surgery, Department of Surgery, Stanford University, CA, USA

**Keywords:** Breast implants, Pectoralis major, Latissimus dorsi muscle, Botox, Pain, Animation deformity

## Abstract

**Introduction:**

Breast reconstruction is most commonly performed using implant-based reconstruction. Patients with subpectoral implant placement with or without latissimus dorsi (LD) muscle coverage can experience muscle pain and animation deformity. Due to minimal literature describing the use of botulinum toxin (BTX-A) treatment for these side effects from implant-based reconstruction, we report our outcomes.

**Methods:**

A retrospective chart review of breast reconstructive patients for a single surgeon was performed. Patients who underwent BTX-A injection for muscular pain, spasm, or animation deformity were identified and outcomes reviewed. They were also stratified based on radiation treatment and type of muscle flap used.

**Results:**

Eleven patients were identified who had a submuscular pectoralis pocket and/or a pedicled latissimus dorsi flap. Nineteen breasts were treated. The average amount of time from the patient’s last surgery to BTX-A injection was 11.2 months. 25–100 units were used per injection with an average of 60 units. Non-irradiated patients had signifycantly lower post-injection capsular contracture Baker grades and significantly lower amounts of BTX-A were injected. Patients who had both pectoralis major muscle and LD implant-reconstruction were significantly less likely to have improvement in pain/tightness. Most patients reported improvement or resolution of their pain and/or animation deformities.

**Conclusion:**

Implant-based reconstruction using the pectoralis major and/or LD muscles can be plagued with muscular pain, spasm, and animation deformities. The use of BTX-A is a diagnostic and therapeutic modality for these post-breast reconstruction patients with most patients having resolution of symptoms without the need for additional surgery.

## 1. Introduction

Botulinum toxin type A (BTX-A) is a powerful tool. It inhibits acetylcholine and substance P release causing muscle paralysis with a less clear mechanism of action causing decreased pain [1, 2]. One realm BTX-A has shown particular success in is pain management, with recognized applications ranging from migraines to pelvic floor dysfunction [3]. In the plastic surgeon’s armamentarium, BTX-A has an established role with respect to enhancing cosmesis and improving functionality [4]. It has had an increasing role in post-operative management of women who undergo breast reconstruction, specifically implant-based. While this is the most common type of breast reconstruction performed, it can be associated with significant post-operative muscle pain and dysfunction. Subpectoral implant placement, whereby the tissue expander or implant is placed beneath the pectoralis major, has been the decades’ long gold standard in implant-based breast reconstruction [5]. Despite the resurgence of prepectoral breast reconstruction, many patients are poor candidates for such, either due to mastectomy flap thickness or availability of tissue, especially in thin patients, for subsequent fat grafting to help camouflage the transition between the implant and chest wall [6]. Relative to prepectoral reconstructtion, submuscular implantation supports reduced risk of capsular contracture, contour deformity, and mastectomy flap necrosis [7]. The tradeoff incurred is the increased risk of muscular spasms and animation deformity, whereby the implant is displaced superolaterally whenever the overlying muscle contracts [8].

Animation deformity can range in severity, from slight discomfort and embarrassment to profound pain and restriction of physical function. It is estimated that at least 75% of subpectoral patients experience some degree of AD, and it is a common cause for revision surgery, with approximately 28% of patients pursuing reoperation [8–10]. Pectoral-sparing approaches do not always avoid these complications either; pedicled latissimus dorsi reconstruction is commonly associated with animation deformity with an incidence as high as 100% in the literature [11, 12]. Some surgeons have taken to prophylactically denervating the thoracodorsal nerve during latissimus reconstruction, but this approach can be challenging and is not always successful [11, 13, 14]. Therapies offered for patients suffering from animation deformity or muscle spasm include conversion to a prepectoral position, division of the pectoralis major muscle, and selective nerve ablation [15, 16]. However, all revision modalities warrant a return to the operating room, which is associated with increased time, cost, and morbidity. For patients suffering from such complications and are reticent to return to the operating room, studies suggest BTX-A may be of benefit [12, 17–20]. While the effect of BTX-A can be considered temporary, injection of it into the offending muscle stands as a low-cost, low-risk alternative that can be performed on an outpatient basis. There exists limited data regarding the use of BTX-A in post-implant-based breast reconstruction pain and deformity [12, 20]. In this series, we examined the utility of BTX-A therapy for patients who had undergone submuscular implant-based breast reconstruction with either pectoralis major and/or latissimus muscle coverage who develop post-operative muscular pain and/or animation deformity.

## 2. Methods

A retrospective chart review of patients undergoing breast reconstruction with a single surgeon was performed. Patients who underwent implant-based reconstruction with supplemental use of the pectoralis major muscle and latissimus dorsi muscle were identified. Those who had persistent post-operative muscular pain, contraction, or animation deformity and underwent BTX-A injection for these symptoms and signs were identified. Their demographic information was reviewed along with the details surrounding the BTX-A injection (location, amount, number of treatments), as well as the patients’ outcomes. Improvement of muscular pain and animation deformity were the primary endpoints used to determine the need for repeat injection or surgery. This was determined based on the patient’s report, as well as on physical exam. The study was approved by the institutional review board.

### 2.1 Procedure

**Figure 1:**
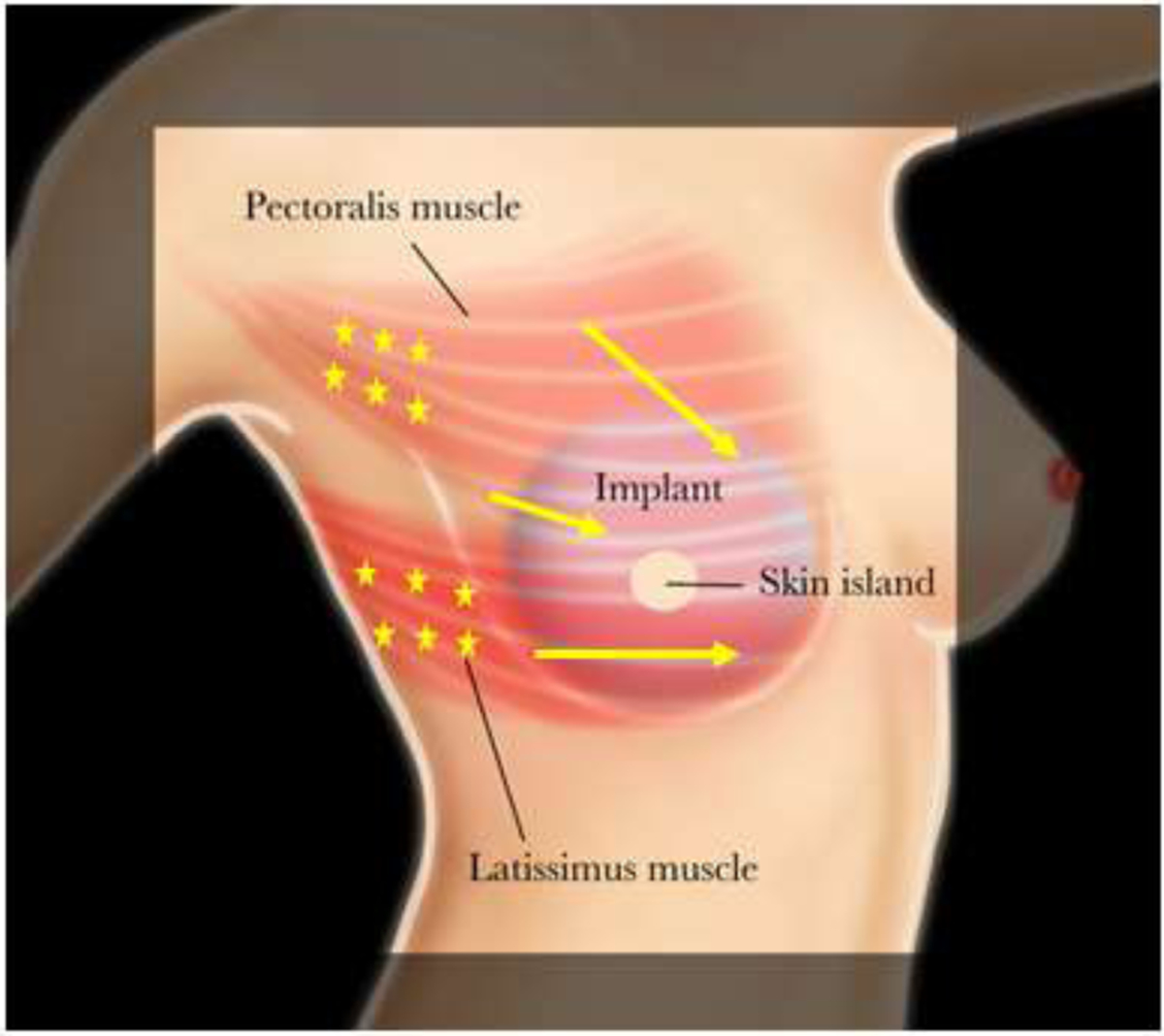
Botulinum toxin A injection into the pectoralis major and/or latissimus dorsi muscles were targeted based on the location of the patient’s pain and the muscle origin in increments of 0.2 mL (4 units) per site. Stars indicate the location of injections.

Botulinum toxin type-A injection was performed in an outpatient clinic without any sedation or local anesthetic. The freeze-dried 100 unit BTX-A vial was reconstituted with 5 mL of sterile sodium chloride (0.9%). The location of the patient’s pain and/or muscle tightness was identified at rest and with activation by having the patient place their fists on their hips while adducting and internally rotating their arms. Occasionally focal areas of muscle spasms can be seen visually, as well as by palpation. In addition, the patient’s reported sites of pain and/or tightness were marked to provide targeted injection including the pectoralis major muscle’s origin (Figure 1). The amount of BTX-A that was administered depended on the severity of the patient’s pain and/or animation deformity. Injections of 4 units/0.2ml BTX-A doses were performed at 2cm intervals using a 30G ¼ inch needle across the affected muscle without image-guidance.

The areas previously marked during the exam identifies the areas of injection, which are prepped with alcohol. The pectoralis major muscle is palpated under the skin and confirmed with muscle activation. The skin and muscle at the site of the injection is pinched between fingers and the implant is displaced away from the injection site. The injection is performed using the entire length of the 30G ¼ inch needle into the tissue isolated between the fingers. Awareness of the injection location and length of the injecting needle, as well as displacement of the implant was performed to avoid injury to the underlying implant. Of note, the use of BTX-A for these purposes is off-label and not yet approved by the FDA.

### 2.2 Statistical analyses

All statistical analyses were completed using Stata v.15 (StataCorp, College Station, TX). The Shapiro-Wilk test was used to determine whether continuous variables were normally distributed. Clinical data were compared between cohorts using Kruskal-Wallis and Fisher’s Exact analyses. The two-tailed threshold for statistical significance was set at an alpha value of 0.05.

## 3. Results

From 2012 to 2019, eleven women meeting inclusion criteria were identified. The average age was 51.5 years (range 34–73 years old) with the majority of patients (9, 81.8%) having a diagnosis of breast cancer (Table 1). All patients had a submuscular pectoralis pocket and/or a pedicled latissimus dorsi flap present with 4 patients undergoing unilateral implant-based breast reconstruction. Six patients had submuscular pectoralis implant placement alone, 4 patients had a pedicled latissimus dorsi flap in conjunction with subpectoral placement of implants, and 1 patient having a pedicled latissimus only with an implant. A total of 19 breasts were treated. Ten patients presented with tight muscle spasms and pain with 3 patients also reporting animation deformity and 1 patient having animation deformity only. One patient experienced a tear of the latissimus dorsi muscle attachment resulting in acute onset of pain. The remaining patients had progressively worsening muscular pain with 3 patients having a history of infection, 1 patient having a hematoma requiring evacuation, and the remaining with an unremarkable peri-operative course. The average amount of time from the patient’s last surgery to BTX-A injection was 11.2 months (range 1.6–25.5 months). The average amount of BTX-A used per injection site was 60 units (range 25–100 units) with the total median number of units used for the pectoralis major muscle and latissimus dorsi muscle being 50 and 100 units, respectively.

Patient response to the BTX-A injections were assessed during their post-operative visits. The average length of follow-up was 8.5 months (range 8 days to 24.5 months) from the time of the patient’s last BTX-A injection. Two patients were lost to follow up. The majority of remaining patients (6 of 9, 67%) reported improvement or resolution of their pain and/or animation deformities, as well as having softening of their capsule contracture to grade 1–2 on exam. Three patients required 2 or more BTX-A injections and 1 patient had denervation of bilateral pectoral nerves due to the severity of the animation deformity that had improved, but recurred, with BTX-A. No adverse complications were associated with BTX-A use including injection site infection, generalized muscle weakness, breathing difficulties or hypersensitivity reactions. When stratifying patients by their history of radiation therapy, 4 patients were identified as having undergone radiation (Table 2a). All of these patients had symptoms on palpation and contraction, and had tried physical therapy and pain medications prior to the use of BTX-A. When comparing the effect of BTX-A injection on the grade of capsular contracture pre- and post-injection, a statistically significant difference between patients with and without a history of radiation was found. Patients having a history of radiation had less improvement in their capsular contracture compared to non-irradiated patients.

In addition, when analyzing the effects by breast, non-irradiated breasts received significantly lower amounts of BTX-A than irradiated breasts (Table 2b). Patients who were non-irradiated were more likely to have improvement in their symptoms of pain/tightness. Patients were also compared based on the type of muscle coverage used for the implant: pectoralis major muscle only, pectoralis major muscle and latissimus muscle, or latissimus dorsi muscle only (Table 3a). Patients with pectoralis major muscle only had the highest rate of animation deformity. Latissimus dorsi flaps received the greatest amount of Botox. When stratifying breasts by type of muscle cover, breasts having both the pectoralis major muscle and latissimus dorsi flaps were significantly less likely to have improvement in pain/tightness symptoms after injection (Table 3b).

## 4. Discussion

Breast reconstruction is becoming increasingly widespread after mastectomy. The most common approach is implant-based reconstruction with the majority of patients undergoing subpectoral placement versus prepectoral placement [7, 21, 22]. The latissimus muscle is a known adjunct in implant-based reconstruction, especially in patients who have had prior radiation therapy or failed breast reconstruction. The primary drawbacks to using submuscular reconstruction, however, is the potential for muscle spasms and animation deformity. Because of these complications, we sought to evaluate our use of BTX-A for patients who had undergone subpectoral implant placement and/or a pedicled latissimus dorsi muscle flap for breast reconstruction. The majority of patients developed muscle spasms and/or animation deformity without a specific inciting event except for one patient having a traumatic tear of their latissimus dorsi muscle attachment resulting in acute onset of pain. The remaining patients generally had an unremarkable peri-operative course leading to progressively worsening muscular pain, but 3 patients did have a history of infection and 1 patient had a hematoma requiring evacuation. These risk factors have been previously identified as having a high likelihood of causing capsular contracture, which is associated with muscular pain [23]. The majority of patients with follow up reported improved muscular spasm and animation deformity after only one BTX-A injection. There is not a clear explanation for this finding as its effect on skeletal muscle weakness is typically 3–6 months and its impact on pain receptors has not been clearly elucidated [3, 24]. BTX-A can cause muscle fibers to undergo progressive, but reversible atrophy, and eliminate isometric muscle contraction during its effective period with the potential to reduce long-term contraction resulting in an improved outcome [1, 25]. We hypothesize that a single injection can affect the muscle physiology allowing the pectoralis major muscle or latissimus dorsi muscle maintain a more relaxed state in relation to the implant, but further study is needed.

In general, the literature on the use of BTX-A in breast reconstruction is conflicting. A nonrandomized prospective study of 48 patients (22 patients receiving and 26 patients not receiving BTX-A infiltration at the time of mastectomy) reported a significant decrease in post-operative pain and decreased narcotic requirement in the study group [18]. A randomized, prospective study of 30 women undergoing bilateral mastectomies divided into 2 groups (15 patients each) received a total of 2 mL of 40 units of BTX-A toxin or 2 mL of normal saline placebo at the time of subpectoral tissue expander placement [19]. The study reported a statistically significant decrease in pain, increase in volume of expansion per post-operative visit, and overall decrease in narcotic use in the group receiving BTX-A [19]. This is in contrast to a prospective, randomized, double-blinded controlled trial including 23 patients where patients had BTX-A toxin injected on one side and normal saline placebo injected on the other at the time of mastectomy that found no significant differences in pre-operative to post-operative pain scores [26]. A systematic review of the literature found that BTX-A may alleviate post-operative pain associated with the placement of subpectoral tissue expanders and implants; however, the majority of the studies had patients receiving intra-operative BTX-A injections (91.8%) with only 3.5% of women receiving post-operative injections [17]. The two studies that reported post-operative BTX-A injections in women undergoing breast reconstruction with latissimus flaps and subpectoral implants reported subjective pain improvement or alleviation of muscle spasms [12, 20]. Given the paucity of data about post-operative use, we feel that our findings can influence management for these patients.

Our results showed that non-irradiated patients had significantly lower Baker grades of capsular contracture post-injection compared to irradiated patients. The non-irradiated breasts also were significantly more likely to demonstrate improvement in symptoms of pain/tightness. This suggests that BTX-A is more efficacious in treating capsular contracture among non-irradiated patients. In addition, non-irradiated patients received a significantly lower amount of BTX-A. These results indicate that the skin and soft tissue changes after radiation may have a negative impact on the effectiveness of BTX-A. When comparing the type of muscle coverage using pectoralis major muscle and/or latissimus dorsi muscle, the combined use of these muscles were significantly less likely to have an improvement in pain/tightness symptoms after injection and a higher post-injection pain score. With muscle being used from two sites, it is not surprising that patients would be more symptomatic as it is likely there is an additive effect when combining both muscles. The limitations associated with this study are its small sample size, retrospective nature, and variable length of follow-up. In addition, while the use of BTX-A has been reported in implant-based reconstruction, a specific methodology still needs to be established. Approaches have ranged from the number of units used to the number and specific locations of the injections, and the optimal dose of BTX-A has yet to be determined.

## 5. Conclusions

The use of BTX-A can be a diagnostic and therapeutic modality for implant-based breast reconstruction patients who experience muscular pain, spasm, and animation deformities due to the manipulation of their pectoralis major and/or latissimus dorsi muscles. This study supplements the available literature that generally supports the use of BTX-A for these symptoms and may negate the need for additional surgery in these patients. Therefore, botulinum toxin A is a promising first-line therapy for post-implant breast reconstruction patients experiencing muscular pain and dysfunction.

## Figures and Tables

**Table 1:** Demographics and patient characteristics.

Characteristics	No. of patients
Diagnosis:	
• History of breast cancer	9
• Genetic predisposition	3
Type of reconstruction:	
• Bilateral	7
• Unilateral	4
Involved sites:	
• Pectoralis major muscle only	6
• Pectoralis major + Latissimus dorsi muscle	4
• Latissimus dorsi muscle only	1
Signs/Symptoms:	
• Muscle spasm/tightness only	7
• Muscle spasms + Animation deformity	3
• Animation deformity only	1

**Table 2a:** Clinical Characteristics and Outcomes of Patients, Stratified by History of Radiation (n=11 patients).

Characteristic	History of Radiation (n=4)	No History of Radiation (n=7)	p-value
Presence of Animation Deformity – n (%)	0 (0)	4 (57)	0.19
Duration of Symptoms (Months) (IQR)	8 (7)	8 (13)	0.51
Type of Prior Therapy – n (%)			
Physical Therapy	4 (100)	7 (100)	1.0
Pain Medications	4 (100)	6 (86)	0.43
Muscle Relaxants	2 (50)	4 (57)	0.82
Severity of Symptoms – n (%)			
Visible	1 (25)	4 (57)	0.30
On Palpation	4 (100)	6 (86)	0.43
On Contraction	4 (100)	6 (86)	0.43
Length of Time to Botox Injection (Months) (IQR)	8 (11)	8 (8)	0.71
Amount of Botox Units Injected (IQR)	75 (50)	50 (10)	0.26
Pre-Injection Capsular Contracture Grade (IQR)	3 (0)	2 (1)	0.13
Pre-Injection Pain Score (IQR)	9 (1)	8 (4)	0.45
Time to Improvement (Weeks) (IQR)	4 (1)	3 (1)	0.78
Duration of Symptomatic Relief (Months) (IQR)	11 (15)	10 (29)	0.27
Post-Injection Capsular Contracture Grade (IQR)	2 (1)	1 (1)	**0.05**
Post-Injection Pain Score (IQR)	4 (4)	2 (4)	0.27
Improvement in Pain/Tightness	2 (50)	7 (100)	0.11
Improvement in Animation Deformity*	n/a	4 (100)	n/a

IQR: interquartile range

* proportions calculated out of the number of patients who had animation deformity

**Table 2b:** Clinical Characteristics and Outcomes of Patients by Breast, Stratified by History of Radiation (n=19 breasts).

Characteristic	History of Radiation (n=5)	No History of Radiation (n=14)	p-value
Presence of Animation Deformity – n (%)	0 (0)	7 (50)	0.30
Duration of Symptoms (Months) (IQR)	9 (6)	8 (13)	1.0
Type of Prior Therapy – n (%)			
Physical Therapy	5 (100)	14 (100)	1.0
Pain Medications	5 (100)	12 (86)	1.0
Muscle Relaxants	3 (60)	8 (57)	1.0
Severity of Symptoms – n (%)			
Visible	1 (20)	6 (43)	0.6
On Palpation	5 (100)	10 (71)	0.53
On Contraction	5 (100)	10 (71)	0.53
Length of Time to Botox Injection (Months) (IQR)	10 (13)	8 (8)	0.68
Amount of Botox Units Injected (IQR)	100 (50)	50 (25)	0.04
Pre-Injection Capsular Contracture Grade (IQR)	3 (2)	2 (1)	0.10
Pre-Injection Pain Score (IQR)	8 (1)	8 (4)	0.46
Time to Improvement (Weeks) (IQR)	4 (1)	3 (1)	0.85
Duration of Symptomatic Relief (Months) (IQR)	12 (15)	14 (29)	0.23
Post-Injection Capsular Contracture Grade (IQR)	2 (0)	1 (1)	0.05
Post-Injection Pain Score (IQR)	2 (3)	2 (4)	0.18
Improvement in Pain/Tightness	3 (60)	14 (100)	0.05
Improvement in Animation Deformity	n/a	7 (100)	n/a

IQR: interquartile range

n/a: not applicable

**Table 3a:** Clinical Characteristics and Outcomes of Patients, Stratified by Muscle Coverage Type (n=11 patients).

Characteristic	Pectoralis (n=6)	Pectoralis + Latissimus (n=4)	Latissimus Only (n=1)	p-value
Presence of Animation Deformity – n (%)	6 (100)	0 (0)	0 (0)	0.06
Duration of Symptoms (Months) (IQR)	11 (15)	8 (5)	5 (n/a)*	0.62
Type of Prior Therapy – n (%)				
Physical Therapy	6 (100)	4 (100)	1 (100)	1.0
Pain Medications	5 (83)	4 (100)	1 (100)	1.0
Muscle Relaxants	3 (50)	2 (50)	1 (100)	1.0
Symptom Severity – n (%)				
Visible	4 (67)	1 (25)	0 (0)	0.4
On Palpation	5 (83)	4 (100)	1 (100)	1.0
On Contraction	5 (83)	4 (100)	1 (100)	1.0
Length of Time to Botox Injection (Months) (IQR)	10 (10)	9 (8)	5 (n/a)	0.62
Amount of Botox Injected (IQR)	50 (25)	75 (50)	100 (n/a)	0.07
Pre-Injection Capsular Contracture Grade (IQR)	3 (1)	3 (0)	0 (n/a)	0.38
Pre-Injection Pain Score (IQR)	8 (4)	9 (1)	9 (n/a)	0.38
Time to Improvement (Weeks) (IQR)	4 (1)	4 (1)	2 (n/a)	0.33
Duration of Symptomatic Relief (IQR)	14 (29)	7 (11)	36 (n/a)	0.23
Post-Injection Capsular Contracture Grade (IQR)	2 (1)	2 (1)	0 (n/a)	0.24
Post-Injection Pain Score (IQR)	2 (3)	4 (3)	0 (n/a)	0.12
Improvement in Pain/Tightness – n (%)	6 (100)	2 (50)	1 (100)	0.29
Improvement in Animation Deformity – n (%)	6 (100)	n/a	n/a	n/a

IQR: interquartile range

* n/a: not applicable

**Table 3b:** Clinical Characteristics and Outcomes of Patients by Breast, Stratified by Muscle Coverage Type (n=19 breasts).

Characteristic	Pectoralis (n=12)	Pectoralis + Latissimus (n=5)	Latissimus Only (n=2)	p-value
Presence of Animation Deformity – n (%)	8 (67)	0 (0)	0 (0)	0.07
Duration of Symptoms (Months) (IQR)	11 (15)	8 (5)	5 (19)	0.97
Type of Prior Therapy – n (%)				
Physical Therapy	12 (100)	5 (100)	2 (100)	1.0
Pain Medications	10 (83)	5 (100)	2 (100)	1.0
Muscle Relaxants	6 (50)	2 (40)	2 (100)	0.34
Symptom Severity – n (%)				
Visible	6 (50)	1 (20)	0 (0)	0.34
On Palpation	9 (75)	5 (100)	2 (100)	0.57
On Contraction	9 (75)	5 (100)	2 (100)	0.57
Length of Time to Botox Injection (Months) (IQR)	10 (10)	9 (8)	5 (21)	0.95
Amount of Botox Injected (IQR)	50 (18)	75 (50)	100 (0)	0.08
Pre-Injection Capsular Contracture Grade (IQR)	2 (1)	3 (0)	0 (3)	0.13
Pre-Injection Pain Score (IQR)	8 (4)	9 (1)	9 (2)	0.24
Time to Improvement (Weeks) (IQR)	4 (1)	4 (1)	2 (1)	0.13
Duration of Symptomatic Relief (IQR)	14 (29)	7 (11)	36 (24)	0.15
Post-Injection Capsular Contracture Grade (IQR)	2 (1)	2 (1)	0 (2)	0.24
Post-Injection Pain Score (IQR)	2 (3)	4 (3)	0 (2)	0.06
Improvement in Pain/Tightness – n (%)	12 (100)	2 (40)	2 (100)	0.05
Improvement in Animation Deformity – n (%)	8 (100)	n/a	n/a	n/a

IQR: interquartile range
